# Efficacy and safety of danoprevir plus sofosbuvir in GT 1, 2, 3, or 6 chronic hepatitis C patients with or without cirrhosis in China

**DOI:** 10.1097/MD.0000000000026312

**Published:** 2021-06-18

**Authors:** Shufang Pan, Kai Feng, Ping Huang, Yingfu Zeng, Liu Ke, Xiaodong Yang, Jing Liu, Chaoshuang Lin

**Affiliations:** a Department of Infectious Diseases, The Third Affiliated Hospital of Sun Yat-Sen University; b Department of Infectious Diseases, The People's Hospital of Lianjiang, Guangdong Province; c Liuzhou People's Hospital, Liuzhou, Guangxi; d Kunming Third People's Hospital, Kunming, China.

**Keywords:** hepatitis C, ritonavir-boosted danoprevir, sofosbuvir, sustained virologic response

## Abstract

All-oral direct-acting antiviral therapies are becoming the choice for hepatitis C (HCV) treatment. In this study, we aimed to evaluate the efficacy and safety of ritonavir-boosted danoprevir (DNVr) plus sofosbuvir±ribavirin on HCV genotype 1, 2, 3, or 6 in the real world in China.

In this observational, prospective, multicenter cohort, we enrolled a total of 58 patients with HCV genotype 1, 2, 3, or 6 patients from July 2018 to December 2019. All patients were treated with DNVr plus sofosbuvir ± ribavirin for 12 weeks and then followed up for 12 weeks. The primary endpoint was the rate of sustained virologic response at week 12 after the end of treatment (SVR12). The secondary endpoint was virologic response rate at end-of-treatment and adverse event outcome.

Of the 58 patients who were enrolled, 5.2% (n = 3) had genotype 1a; 43.1% (n = 25) had HCV genotype 1b; 17.2% (n = 10) had genotype 2a; 5.2% (n = 3) had genotype 3a; 8.6% (n = 5) had genotype 3b; and 20.7% (n = 12) had genotype 6a. The virologic response rate at end-of-treatment was 100% (58/58). The HCV-RNA results of 5 patients were absent at week 12 after treatment. Among the 53 patients, SVR12 rate achieved 100% (53/53) with DNVr plus sofosbuvir ± ribavirin treatment in patients with HCV genotype 1b, 2a, 3, and 6a. For compensated cirrhosis and noncirrhosis patients, SVR12 was 100% with DNVr plus sofosbuvir ± ribavirin treatment. No serious event was observed during the treatment and follow-up. Only 5 patients had mild adverse events.

DNVr plus sofosbuvir ± ribavirin for 12 weeks provided 100% SVR12 in a broad patient population and were well tolerated, which may be a promising regimen for CHC treatment.

## 1 Introduction

The number of patients with infected hepatitis C virus (HCV) is estimated to be about 10 million in China and the incidence rate is increased year by year.^[1]^ Cirrhosis and hepatocellular carcinoma are the leading causes of death in patients with chronic hepatitis C.^[1]^ The most prevalence of HCV genotype in China was 1b, accounting for 56.8% and followed by 2a, 3b, 6a, and 3a.^[2,3]^ The discovery of direct anti-viral agents (DAAs) revolutionized the treatment landscape of HCV. Moreover, oral combination DAAs largely improved the sustained virologic response rate (SVR) to render HCV a routinely curable disease.^[4]^

Danoprevir (DNV) is a new generation of HCV NS3/4A protease inhibitor and preclinical study demonstrated that DNV had biochemical potency against HCV genotype 1, 2b, 3a, 4, 5, and 6.^[5]^ DNV has been approved for non-cirrhotic patients with HCV genotype 1b infection in combination with peginterferon and ribavirin. Ritonavir-boosted DNV (DNVr) plus peginterferon alfa-2a/ribavirin achieved 97% SVR in Chinese patients with genotype 1b without cirrhosis and achieved 91.7% SVR in Asian patients with genotype 1b infection with cirrhosis.^[6]^ All-oral combination of NS5A inhibitor ravidasvir, DNVr and ribavirin revealed the SVR12 rates of 99% and were well tolerated in treatment-naïve, noncirrhotic HCV Chinese patients with genotype 1.^[7,8]^

Sofosbuvir is a HCV nucleotide analog NS5B polymerase inhibitor, which characterized the *in vitro* antiviral activity against genotypes 1 to 6.^[9,10]^ Sofosbuvir has been approved for the treatment of HCV genotype 1 to 6 infection in combination with ribavirin ± peginterferon, NS5A inhibitor ledipasvir or velpatasvir by China food and drug administration center. A phase 3, randomized study demonstrated that sofosbuvir in combination with HCV NS3/4 protease inhibitor simeprevir for 12 weeks produced the SVR of 97% for HCV genotype 1-infected patients without cirrhosis.^[11]^

DNVr in combination with peginterferon has been developed for HCV treatment.^[1,6]^ However, injection requirement of peginterferon-based treatment bring inconvenience for HCV patients. For the all-oral era of HCV treatment, we assessed the efficacy of DNVr in combination with ravidasvir in treatment-naïve noncirrhotic HCV genotype 1 patients.^[8]^ Besides, HCV NS5A inhibitor daclatasvir has low barrier to resistance mutations.^[12]^ Thus, combination of DNVr plus sofosbuvir ± ribavirin may serve as a potent pan-genotypic regimen against HCV infection with higher efficacy. To our knowledge, this real-world prospective study is the first to evaluate the efficacy and safety of 12-week treatment regimen of DNVr plus sofosbuvir ± ribavirin in the HCV genotype 1, 2, 3, and 6-infected patients with or without cirrhosis in China.

## 2 Patients and methods

### 2.1 Study design and patients

This is a prospective, multicenter study to evaluate the efficacy and safety of DNVr plus sofosbuvir ± ribavirin for HCV-infected patients in multiple hospitals including the Third Affiliated Hospital of Sun Yat-Sen University, the People's Hospital of Lianjiang, Third People's Hospital of Kunming City and Liuzhou's People's Hospital. Eligible subjects included HCV-infected patients aged 18 years or older who were prescribed with DNVr plus sofosbuvir ± ribavirin from July 2018 to December 2019. Whether ribavirin was used or not was according to the discretion of clinical physicians. This cohort also included patients with syphilis infection, diabetes, or carcinoma. Patients were excluded if they had decompensated cirrhosis or hepatitis B virus infection.

All patients received DNVr 100 mg/100 mg twice daily, 400 mg of sofosbuvir once daily and/or ribavirin at 1000 mg (<75 kg) or 1200 mg (≥75 kg) daily for 12 weeks. Then all patients were followed for additional 12 weeks. The primary endpoint was the percentage of patients with SVR12. The secondary endpoint was the virologic response rate at end-of-treatment (EOT) and adverse event outcome.

This protocol was approved by local Institution Review Committees and all patients provided written informed consent before enrolment. This study was performed according to Declaration of Helsinki.

### 2.2 Clinical assessment

Demographic variables included age, sex, weight, body mass index, and comorbidities (including diabetes, carcinoma). Routine laboratory tests included alpha fetoprotein, alanine transaminase, aspartate amino transferase, creatinine clearance, white blood cell count, haemoglobin, platelet, HCV genotype and HCV RNA level. Cirrhosis was established on a liver stiffness measurement > 14.6 kPa by Fibroscan or liver biopsy indicating Metavir fibrosis 4. HCV RNA levels were measured at baseline, week 12 at EOT and 12 weeks after treatment completion. Adverse events were recorded during the treatment period and 12-week follow-up.

### 2.3 2Statistical analysis

Normally distributed quantitative variables were expressed as mean ± standard deviation. Categorical variables were expressed as frequencies and percentages. Statistical analyses were performed on SPSS software (version 26, SPSS Inc, Chicago, IL).

## 3 Results

### 3.1 Patient population

In total, 58 patients were enrolled across 3 centers in southern China: 53.4% (n = 31) patients were men with mean age 45 ± 12 years. Among the 58 patients, 5.2% (n = 3) with genotype 1a; 43.1% (n = 25) with HCV genotype 1b; 17.2% (n = 10) with genotype 2a; 5.2% (n = 3) with genotype 3a; 8.6% (n = 5) with genotype 3b; and 20.7% (n = 12) had genotype 6a. The results are similar with the national epidemiology research. Four patients were classified as having compensated cirrhosis. Characteristics of patients are shown as Table 1. Three patients were drug abusers. One patient had esophagus cancer and 1 patient infected with syphilis.

**Table 1:** Baseline demographic and disease characteristics.

Characteristics	N
All patients	58
Male sex, n (%)	31 (53.4%)
Age, y	45 ± 12
Body weight, kg	60 ± 9
Body mass index, kg/m^2^	22 ± 4
Cirrhosis, n (%)	4 (6.9%)
Alpha fetoprotein, ng/ mL	4.6 ± 2.5
ALT, U/L	66 ± 45
AST, U/L	55 ± 43
White blood cell count, × 10^9^ cells/L	6.4 ± 2.0
Hemoglobin, g/L	142 ± 19
Platelet count, × 10^9^/L	218 ± 74
HCV genotype, n (%)	
1a	3 (5.2%)
1b	25 (43.1%)
2a	10 (17.2%)
3a	3 (5.2%)
3b	5 (8.6%)
6a	12 (20.7%)
HCV RNA, IU/mL	4.22 × 10^6^

### 3.2 Efficacy

All 58 patients completed 12-week treatment of DNVr plus SOF ± RBV and the response rate at the end of treatment (EOT) was 100% (58/58). All patients were followed for additional 12 weeks but 3 patients were lost to follow-up and 2 patients have not completed the 12-week follow-up when we performed the data analysis. SVR12 was achieved 100% (53/53) for all 53 HCV patients.

### 3.3 Adverse events

Treatment-related adverse effects were evaluated in 58 patients. There was no serious adverse event during the treatment and follow-up. Only 5 mild adverse events were reported during treatment. There was 1 patient with headache, 1 with heart rate increase, and 1 with diarrhea. A 49-year-old woman developed gross hematuria, leukocytosis, and positive urinary occult blood after taking the drug for one week. HCV medication was not discontinued. Considering urinary tract infection, no anti-inflammatory medication was used and hematuria improved after 24 hours. One patient had hemolytic anemia which may be related with the use of ribavirin and symptom was relieved by dose reduction of ribavirin. No patient discontinued because of adverse effects. Adverse events are described in Table 2.

**Table 2:** Adverse events.

Characteristic	N (%)
Deaths, n (%)	0
Patients with ≥1 serious AE, n (%)	0
Incidence of individual AEs, n (%)	5 (9.4%)
Headache, n (%)	1 (1.9%)
Heart rate increase, n (%)	1 (1.9%)
Transient hematuria, n (%)	1 (1.9%)
Diarrhea, n (%)	1 (1.9%)
Hemolytic anemia, n (%)	1 (1.9%)

### 3.4 Drug-drug interaction

Before patients were enrolled in this study, drug–drug interaction risks were evaluated according to the comorbidities of patients based on the database developed by University of Liverpool (available at hep-druginteractions.org/checker). One patient took a simvastatin to control blood lipid levels and 1 patient took calcium antagonist nifedipine to control blood pressure. Because ritonavir could increase the blood concentration of both simvastatin and nifedipine, the medication for these 2 patients was switched to rosuvastatin and irbesartan, respectively. There was one 45 years’ old male who had esophagus cancer and took capecitabine for carcinoma treatment. One male patient was 41 years’ old and had been taking propranolol for hypertension treatment. No drug–drug interaction-related adverse reactions were observed during the study period.

## 4 Discussion

HCV is classified into 6 different genotypes and high diversity could be observed in China.^[13,14]^ All-oral pan-genotypic DAAs have dramatically simplified the HCV treatment with improving effectiveness and accessibility.^[15]^ Preclinical studies of DNV for HCV demonstrated that the 50% inhibitory concentrations (IC50) of genotype 1a, 1b, 2b, 3a, 6 was 0.20 nmol/L, 0.23 nmol/L, 1.6 nmol/L, 3.5 nmol/L, 0.45 nmol/L, respectively, supporting the clinical investigation of DNV.^[5]^ This is the first multicenter prospective cohort study of real-world clinical settings to assess the efficacy and safety of DNVr plus SOF ± RBV with activity against HCV genotypes 1, 2, 3, or 6 infectious patients with compensated cirrhosis or without cirrhosis. Our study documented that 12-week treatment regimen of DNVr plus SOF ± RBV achieved 100% SVR12 rate. Besides, DNVr plus SOF ± RBV was well tolerated.

In our study, SVR12 rate was not affected by HCV genotype or liver stiffness status. The most common HCV genotype in our study was 1b, which is consistent with previous report of the prevalence in China.^[1]^ Beside, HCV genotype 2, 3, and 6 were also included in this study. DNVr plus SOF ± RBV achieved 100% of SVR12 rate for HCV genotype 1, 2, 6 infected patients and the genotype 3 infection which is considered the most difficult-to-cure genotype. For genotype 1 infection, our results were consistent with previous study that all-oral DNVr plus ravidasvir and ribavirin achieved 99% SVR12 rate in patients with HCV genotype 1.^[7,8]^ Pan-genotypic drug has been recommended for HCV infection by World Health Organization, The American Association for the Study of Liver Diseases and the Infectious Diseases Society of America Present and European Association for the Study of the Liver.^[13,16,17]^ In 2 phase 3 studies, sofosbuvir plus velpatasvir showed the SVR12 rate of >99% for patients infected with HCV genotype 1, 2, 4, 5, or 6.^[18,19]^ In the phase 3study, SVR12 rate was only 95% for HCV genotype 3 after treatment of sofosbuvir and velpastasvir,^[19]^ which is lower than that in our study (100%, 8/8). In the real-world study conducted in the United States, sofosbuvir and velpatasvir achieved the 94.4% of SVR rate for HCV genotype 2 patients and 92.0% of SVR rate for genotype 3.^[20]^ DNVr plus SOF ± RBV provided the 100% of SVR12 rate in patients with compensated cirrhosis in this study. Sofosbuvir–velpatasvir could achieve the 99% of SVR12 rate in patients with cirrhosis.^[18]^ In a word, all-oral DNVr plus SOF ± RBV in the present study showed higher SVR12 rate with 100% for HCV genotype 1, 2, or 6 infected patients with cirrhosis or noncirrhosis, Although all patients with GT3 HCV acquired SVR12 in this study, due to the small number of patients enrolled, more clinical treatment data are needed to confirm the efficacy. Thus, combination of DNVr and SOF ± RBV may be a promising regime for the treatment of HCV patients with cirrhosis or noncirrhosis. Three patients treated with interferon ± ribavirin in previous treatment were enrolled, and all achieved SVR12 after 12 weeks of DNVR + SOF ± RBV.

Adverse events observed in this study were mild such as heart rate increase and cough and no patient discontinued due to adverse events. In a phase 2/3 clinical trial evaluating the all-oral DNVr plus ravidasvir and ribavirin in HCV patients in China, mild adverse events such as anemia, upper respiratory tract infection occurred and no treatment-related severe adverse events occurred.^[8]^ In the study of sofosbuvir with velpatasvir in HCV, most common adverse events were fatigue, headache, and nausea, whereas 2% patients reported serious adverse events.^[21]^ Thus, all-oral DNVr plus sofosbuvir ± ribavirin treatment was well tolerated in this study.

There were several limitations in this cohort study. First, as a real-world study, some patients were lost to follow-up only with HCV RNA level at the EOT. Second, the number of enrolled patients is small and large cohort is needed to verify the results. Third, enrolled patients were heterogeneous such as syphilis infection, which provide the valuable insight of the combination regime in the real world.

In conclusion, all-oral DNVr plus sofosbuvir ± ribavirin was effective for HCV genotype 1, 2, 3, or 6 infected patients with or without cirrhosis in the real world study. These data support the combination regime of DNVr plus sofosbuvir for further evaluation in larger cohort study.

## Acknowledgment

The authors thank for patients’ support for this study.

## Author contributions

**Conceptualization:** Ping Huang, Yingfu Zeng, Liu Ke, Xiaodong Yang, Chaoshuang Lin.

**Data curation:** Shufang Pan, Kai Feng, Ping Huang, Jing Liu.

**Formal analysis:** Shufang Pan, Ping Huang.

**Funding acquisition:** Shufang Pan, Ping Huang, Chaoshuang Lin.

**Investigation:** Shufang Pan, Kai Feng, Ping Huang, Yingfu Zeng, Jing Liu, Chaoshuang Lin.

**Methodology:** Ping Huang, Liu Ke.

**Project administration:** Ping Huang.

**Resources:** Ping Huang.

**Software:** Ping Huang, Xiaodong Yang.

**Supervision:** Ping Huang, Liu Ke.

**Validation:** Ping Huang.

**Visualization:** Ping Huang, Liu Ke, Chaoshuang Lin.

**Writing – original draft:** Ping Huang, Yingfu Zeng, Xiaodong Yang, Chaoshuang Lin.

**Writing – review & editing:** Shufang Pan, Chaoshuang Lin.
